# Botulinium toxin, as bridge to transanal pullthrough in neonate with Hirschsprungs disease

**DOI:** 10.4103/0971-9261.43025

**Published:** 2008

**Authors:** S. M. V. Hosseini, H. R. Foroutan, S. Zeraatian, B. Sabet

**Affiliations:** Department of Pediatric Surgery, Shiraz University Medical Science, Shiraz, Iran

**Keywords:** Botulinium toxin, Hirschsprung's disease, neonatal obstruction, pull through operation

## Abstract

**Aims and Objectives:**

The aim of this study is to find easier way of home care while obviating the colostomy before single stage pull through operation.

**Materials and Methods:**

From August 2005 to December 2006, eight cases of neonatal Hirschsprung disease were treated. Mean age 4.5 (2-6) day/old with absent anorectal inhibitory reflex, rectosigmiod disease in Barium enema, positive Acetylcholine esterase (Ache) staining, good response to rectal washout. They underwent botulinium toxin injection (5 unit /kg/quadrant) in four quadrant intrasphincteric. They were followed until pull through operation in 8-10 weeks post injection.

**Results:**

Four of 8 (50%) cases only needed rectal washout for three to five days post injection until pull through operation, two had decrease in number of rectal washouts /day and the remaining two underwent colostomy five days post injection because of no response.

**Conclusion:**

Botulinium toxin injection can help in palliative care in patients with Hirschsprung disease who are waiting for colostomy or definitive pullthrough. It gives an option of eaiser home care for these patients.

Hirschsprung disease (HD) is a common cause of neonatal obstruction. These neonates usually present with abdominal distension, bilious vomiting and delayed passage of meconium.[1] Majority have rectosigmoid aganglionated segment that respond to rectal dilatation and washout for relieving their obstruction. Although single stage pull through is acceptable option for treating the neonates with HD. Many patients undergo delayed surgery due to various reasons. the reasons may be poor general condition, lack of availability of expertise for pullthrough surgery etc. Under these circumstances, conservative management should sometimes be continued until pull through operation.[2] Our aim in this study is to evaluate the effect of botulinium toxin (BT), as an alternative to routine rectal washout for relieving the obstruction, before definitive pull through operation for easier home care.

## MATERIALS AND METHODS

From August 2005 to December 2006, study was conducted in eight neonates with mean age 4.5 (range, two to six days) who referred with abdominal distension, delayed passage of meconium bilious vomiting after birth and they were diagnosed as neonatal HD. Criteria for inclusion were; 1-absent anorectal reflex 2- BE, in favor of recto-sigmoid disease or descending colon 3- good response to rectal washout for relieving the obstruction 4- positive Acetylcholine esterase (Ache) staining in mucosal biopsy.

Our method of treatment was approved by departmental ethics committee (Main pediatric surgery team in our region) and parental consent was taken. All underwent conservative therapy which included; IV antibiotic (metronidazole 10 mg/kg/dose), nasogastric tube, rectal wash out with warm saline three times /day by trained pediatric nurses. They had anorectal manometery before and two weeks after injection by water perfuse system and punch mucosal biopsy for acethylcholinestrase (Ache) staining 2-3 cm above dentate line for determining the presence of ganglion cell just before Botulinium Toxin (BT) injection. Routine abdominal X-ray at presentation and Barium enema (BE) were performed after relieving the obstruction with 24h stopping of rectal washout, before BT injection. Botulinium toxin (Dysport, Ipsen, 500 U/3 ml) was injected intrasphincteric 5 unit /kg/quadrant in (3,6,9,12 o'clock) after manual localization under very brief general anesthesia.

They were observed in NICU for prevention of apnea which might occurred due to centeral effect of BT injection during hospitalization. Patients positive response to BT injection regarded as, drop in baseline resting rectal pressure of 30% or 10 mmHg in anorectal manometery or they developed spontaneous defecation. They were followed to the time of pull through operation by nurse and pediatric surgeon in clinic.

## RESULT

Eight neonate with mean gestational age 37.5 (Range, 36-39 weeks), mean birth weight 2650 (2000-3250 gr) were evaluated. They presented with moderate to severe abdominal distension and bilious vomiting after 48h postdelivery. They had a severe dilated loops in abdominal X ray and narrow rectosigmoid (n=6) and transitional zone in descending colon (n=2) in following BE. Ache stain were positive in eight cases in favor of aganglionosis and all had absent anorectal inhibitory reflex with mean baseline pressure before and two weeks after BT injection (45±2, 30±1), respectively.

Four of the eight (50%) cases were relieved from obstruction after three to five rectal washouts. They needed three to five days of rectal washes after BT injection until spontaneous defecation started. These four had only mild abdominal distension until pull through operation at 8-10 weeks post injection. Two of the eight (37.5%) cases required three times/day rectal washout before and 8-10 days after injection for symptomatic relieve and decreased to once/day until pull through operation in 8-10 weeks post injection. Remaining two cases (1 rectosigmoid, 1 descending colon) did not improve and underwent colostomy in the fifth day post injection. There were no early or late postoperative complications and all patients were followed for 6-12 months with no early complications except persistent post pull through obstructive symptom in two cases (2 rectosigmoid).

## DISCUSSION

Many etiologies has been described for HD but the recent molecular and embryologic studies have shown that the failure of migration, maturation or degeneration of neural cells are the possible causes of aganglionosis and there is absence of non-adrenergic inhibitory nerves also that are required for normal bowel relaxation.[1,2]

Seventy percent of cases referred with neonatal obstruction have been treated by leveling colostomy and pull through operation. Complications of colostomy has made many other invasive techniques like primary perineal one-stage and laparoscopic assisted pull through operations in practice.[3] However majority of these neonates have other medical and surgical problems (sepsis, jaundice, CHD etc) that need to be treated before undergoing pull through operation.[4,5]

Performing one stage pullthrough in a newborn is a major undertaking. It involves considerable degree of operative risk and technical difficulties. Few selected newborns presenting with low general condition require delayed intervention as they are incapable of tolerating a major procedure. These babies are kept on rectal washouts to relive obstruction. Conservative methods give patients the chance of primary pull through operation instead of undergoing primary colostomy. Performing rectal washout daily by mother has the hazard of rectal perforation, patient inconvenience and electrolyte imbalance plus its psychological effect on parents.

Botulinium toxin has safely been used selectively and reversibly to weaken a variety of voluntary muscles and sphincters in both adults and children.[6] Zhonghua *et al*, has used BT in the treatment of rectosigmoid disease. In the first year, all his patients could defecate spontaneously without constipation and abdominal distention.[7] Many of our patients came from rural area that were not complient with prescribed home care and could not afford prolong hospitalization so, we decided to use this method because of its safe profile and adequate BT half life during the waiting period, in order to find an easier way of home care.

Langer *et al*, has injected the botulinium toxin into the internal anal sphincter (IAS) that has produced the same functional result as anal myectomy without permanent sphincter injury. Internal sphincter achalasia has also been treated in recent studies with intrasphincteric botulinium toxin.[8] Minkes *et al*, reported that BT has similar effects to myectomy and if symptoms persist despite fall in resting pressure, a nonsphincteric cause should be ruled out.[9] Resting IAS pressure decreased in all children four to eight weeks after injection[6] and there were no significant differences between the BT injection and control groups with respect to histology evaluation of neuronal size and number, nerve bundle size and number, inflammation or muscle.[10]

Short-term effect of botulinium toxin on HD has been shown in many recent studies but long-erm effect has not been defined yet. In this study, we have used this short effect which plays its part for solving the non relaxation of involved segment. Four of eight patients did not need rectal washout for relieving the obstruction in home care setting and two had significant decrease in number of rectal washout. Family has reported the easy home care by this method of delayed operation.

We had no early or late postoperative complication but the only problem was the cost of BT that we tried to share the drug between those who needed it, therefore total cost of treatment was reduced considerably.

## CONCLUSION

Botulinium toxin injection is an effective method for palliative care in newborn who are not capable of tolerating a major operative procedure. It obviates the need of repeated rectal washouts in these babies.
